# One Patient, Two Uncommon B-Cell Neoplasms: Solitary Plasmacytoma following Complete Remission from Intravascular Large B-Cell Lymphoma Involving Central Nervous System

**DOI:** 10.1155/2014/620423

**Published:** 2014-02-18

**Authors:** Joycelyn Lee, Soo Yong Tan, Leonard H. C. Tan, Hwei Yee Lee, Khoon Leong Chuah, Tiffany Tang, Richard Quek, Kevin Tay, Miriam Tao, Soon Thye Lim, Mohamad Farid

**Affiliations:** ^1^Department of Medical Oncology, National Cancer Centre Singapore, 11 Hospital Drive, Outram Road, Singapore 169610; ^2^Department of Pathology, Singapore General Hospital, 11 Jalan Tan Tock Seng, Singapore 169608; ^3^Department of Pathology, Tan Tock Seng Hospital, Singapore 308433

## Abstract

Second lymphoid neoplasms are an uncommon but recognized feature of non-Hodgkin's lymphomas, putatively arising secondary to common genetic or environmental risk factors. Previous limited evaluations of clonal relatedness between successive mature B-cell malignancies have yielded mixed results. We describe the case of a man with intravascular large B-cell lymphoma involving the central nervous system who went into clinical remission following immunochemotherapy and brain radiation, only to relapse 2 years later with a plasmacytoma of bone causing cauda equina syndrome. The plasmacytoma stained strongly for the cell cycle regulator cyclin D1 on immunohistochemistry, while the original intravascular large cell lymphoma was negative, a disparity providing no support for clonal identity between the 2 neoplasms. Continued efforts atcataloging and evaluating unique associations of B-cell malignancies are critical to improving understanding of overarching disease biology in B-cell malignancies.

## 1. Manuscript

Second lymphoid neoplasm following the diagnosis and treatment of non-Hodgkin's lymphoma (NHL) is a recognized phenomenon. A systematic analysis of national cancer registries involving more than 100000 patients with NHL revealed a 2-3-fold increase in incidence of a second lymphoid neoplasm [1]. The causes for this tendency are diverse, complex, and incompletely understood. While myeloid malignancies, also slightly more common in patients with prior NHL, are known to be induced by cytotoxics like alkylators and anthracyclines used in NHL treatment, second lymphoid cancers are less plausibly attributable to the mutagenic effects of antecedent therapy. The presence of exposures common to the pathogenesis of successive malignancies is one likely underlying factor; for instance, coexistence of immunosuppression and oncogenic viruses like Epstein-Barr virus (EBV) is implicated in cases of EBV-related diffuse large B-cell lymphoma (DLBCL) developing following angioimmunoblastic T-cell lymphoma (AITL) [2]. Similarly, multiple myeloma and chronic lymphocytic leukemia (CLL), which have several analogous biological features (e.g., precursor monoclonal proliferations), have been infrequently associated in individuals [3], again suggesting shared underlying genetic and/or environmental triggers [4]. We describe here a unique case of a plasma cell neoplasm arising following successful treatment of an aggressive B-cell lymphoma.

## 2. Case Presentation

**Figure 1:**
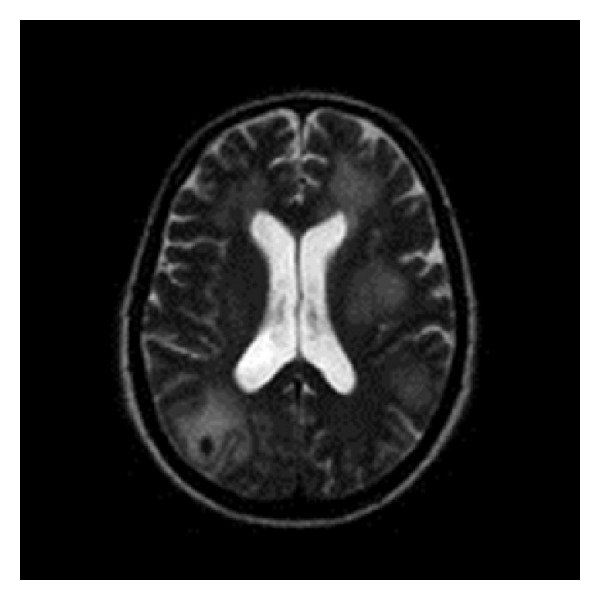
Multiple hemorrhagic foci in bilateral cerebral hemispheres on MRI.

**Figure 2:**
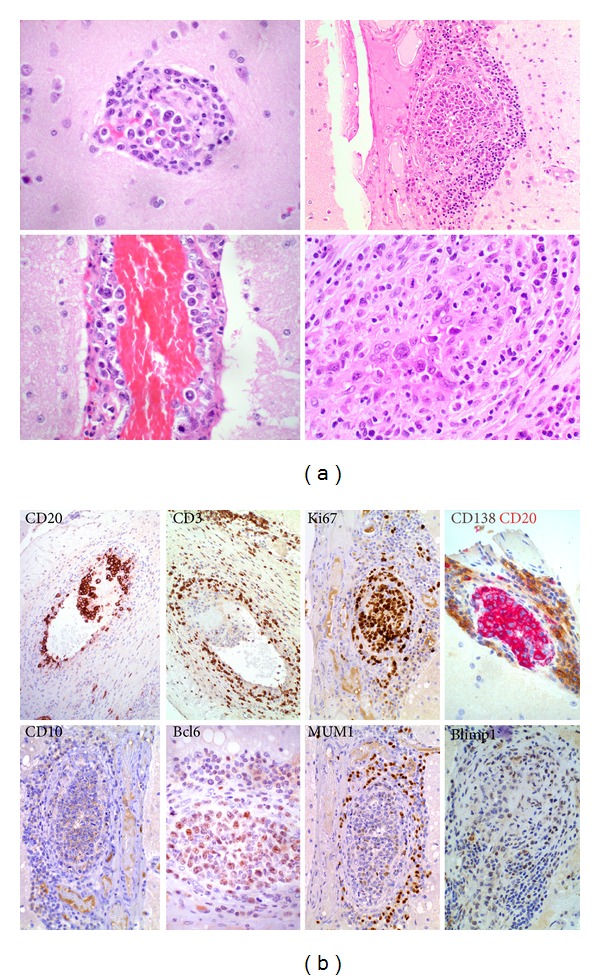
(a) Intravascular large B-cell lymphoma of brain. Hematoxylin and eosin (H/E) staining highlights intravascular large B-cell lymphoma in the brain, featuring large cells with moderate pleomorphism, vesicular chromatin, and prominent nucleoli. (b) Intravascular large B-cell lymphoma of brain. Immunohistochemistry confirms the B-cell lineage of the intravascular large cell lymphoma, being CD20+ CD3− and showing a high proliferation fraction by staining for Ki67. It displays a germinal centre phenotype by Hans' criteria, being CD10− bcl6+ MUM1− and lacks blimp1 expression.

A 59-year-old man of Portuguese descent presented with headaches, confusion, and seizures one week after being started on anticoagulation for an incidentally diagnosed venous thromboembolic event. Magnetic resonance imaging (MRI) of the brain revealed multiple hemorrhagic foci in bilateral cerebral hemispheres associated with edema and mass effect (Figure 1). The anticoagulation was reversed pharmacologically, and he underwent a diagnostic and therapeutic right occipital lobectomy. Histological evaluation of the occipital lobe revealed intraluminal aggregates of lymphomatous large cells strongly positive for the pan B- cell marker CD20 associated with a germinal center immunohistochemical phenotype by Hans criteria [5], with a cell proliferation of 90% by Ki-67 immunolabeling (Figures 2(a) and 2(b)). The diagnosis was intravascular large B-cell lymphoma (IV LBCL). A positron emission tomography (PET) scan showed involvement of the paramedical occipital lobe, pons, vertebrae, sacrum, and spleen with hypermetabolic disease. Bone marrow biopsy was negative for lymphomatous infiltration. Blood investigations revealed elevated lactate dehydrogenase (LDH) but otherwise normal end organ function. Specifically, there was no evidence of splenomegaly, hemolysis, or cytopenias to suggest hemophagocytic syndrome. The globulin fraction was normal, consistent with the absence of any paraproteinemia.

He thus had Stage 4 IV LBCL with central nervous system (CNS) involvement; his international prognostic index (IPI) was 3 (raised LDH, Stage 4 and more than 1 extranodal site of involvement). He was treated with 6 cycles of immunochemotherapy with rituximab, cyclophosphamide, doxorubicin, vincristine, and prednisolone (R-CHOP) given every 3 weeks, in combination with high-dose intravenous methotrexate given on the fourth day of each treatment cycle at a dose of 2 grams/m^2^. This was followed by whole brain radiotherapy given at 30 Grays in 15 fractions. The course of treatment was uneventful; MRI brain and PET following treatment revealed complete radiologic response. He remained physically and functionally stable and maintained regular followup with interval clinical and radiologic evaluations revealing no evidence of recurrence.

**Figure 3:**
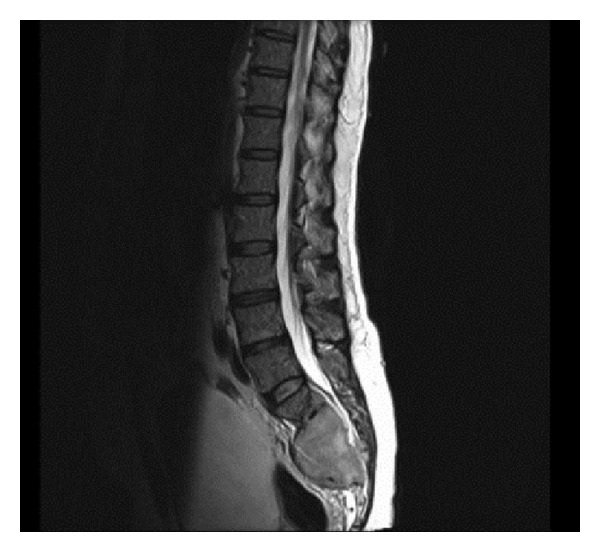
MRI revealing large soft tissue mass in sacral region causing severe canal stenosis and compression of the sacral nerve roots resulting in cauda equina syndrome.

**Figure 4:**
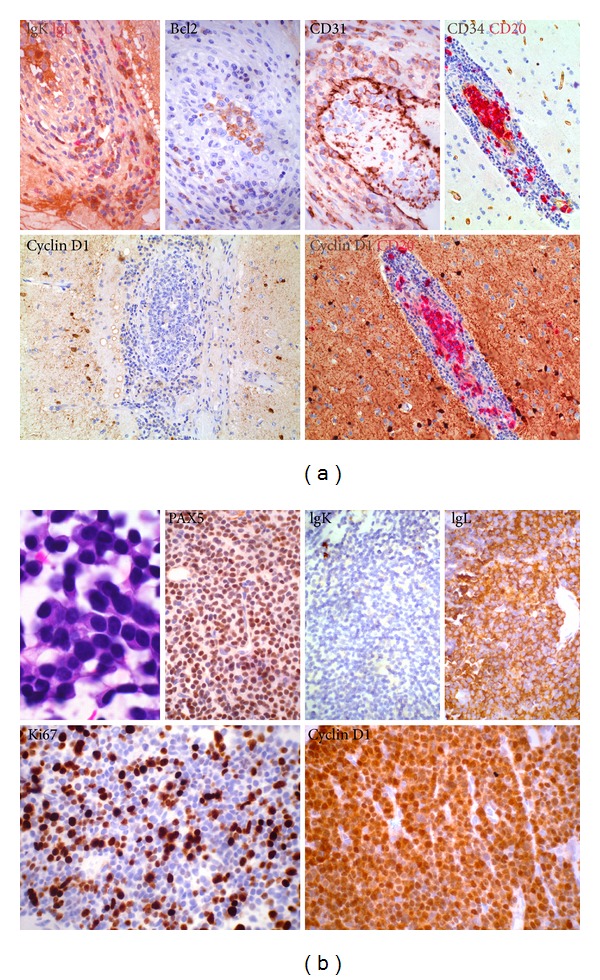
(a) Intravascular large B-cell lymphoma of brain. Neoplastic cells express bcl2 but lack cyclin D1 expression. In situ hybridization shows polytypic immunoglobulin light chain expression in the perivascular reactive plasma cells but not within intravascular neoplastic B cells. (b) Spinal plasma cell neoplasm. Apart from expression of CD138, the spinal plasma cell neoplasm displays expression of PAX5 and cyclin D1 but not CD56. In situ hybridization confirms lambda immunoglobulin light chain restriction.

Two and a half years following initial diagnosis, he presented with a subacute flaccid paraplegia clinically consistent with a cauda equina syndrome. MRI of the spine revealed a 71 by 89 mm soft tissue mass replacing much of the sacrum causing severe canal stenosis and compression of the sacral nerve roots (Figure 3). MRI of the brain showed stable findings of right occipital postsurgical gliosis and postradiation leukoencephalopathy. Computed tomography (CT) scan revealed no evidence of lymphadenopathy or visceral lesions. Histological evaluation of the mass revealed cells displaying uniformly strong expression of CD138, complete negativity for CD20, and uniform strong nuclear expression of cyclin D1, with expression of monotypic lambda immunoglobulin light chain confirming their neoplastic nature (Figures 4(a) and 4(b)).These features excluded recurrent large B-cell lymphoma and were in keeping with terminal plasmacytic differentiation, suggesting the diagnosis of a plasma cell neoplasm. Bone marrow biopsy revealed <5% of singly disposed mature-appearing CD138+ plasma cells, confirmed to be lambda light chain restricted by in-situ hybridization; there was no abnormal karyotype on cytogenetic analysis. Serum and urine immunofixation revealed a monoclonal band detected with anti-lambda, with the monoclonal (M) protein band being 0.4 g/dL. Serum-free light chain analysis was consistent with lambda light chain restriction, with a normal serum kappa-free light chain 3.9 (normal range: 3.3–19.4) mg/L, raised serum lambda-free light chain 171 (5.7–26.3) mg/L, and consequent abnormal serum-free light chain ratio of 0.02 (0.26–1.65). There was no evidence of anemia, renal impairment, hypercalcemia, or lytic bony lesions attributable to the plasma cell dyscrasia.

He thus had evidence of solitary plasmacytoma of bone (SPB) based on the presence of a biopsy-proven solitary plasma cell neoplasm arising from the sacrum associated with systemic features not amounting to smoldering or overt multiple myeloma (monoclonal lambda light chain gammopathy with serum M band <1 g/dL, <10% bone marrow involvement with clonal plasma cells, and absence of associated end organ dysfunction) [6]. This was complicated by cauda equina syndrome. He was continued on high dose steroids with negligible clinical improvement and had been planned for radiotherapy to the sacral mass. Unfortunately, he developed a nosocomial pneumonia during his inpatient stay and passed away before radiotherapy commenced.

Further pathological evaluation of the initial tumor was undertaken to assess for evidence of a clonal relationship between the two tumors. Distinct from the secondary spinal plasmacytoma, the initial cerebral IV LBCL proved to be negative for cyclin D1 expression. Unfortunately, there was insufficient pathological material available for molecular studies to assess the cytogenetics or DNA sequence of the immunoglobulin (Ig) gene.

## 3. Discussion

Our patient demonstrated an excellent response to treatment for his high risk IV LBCL, an aggressive and highly uncommon subtype of large B-cell lymphoma that is characterized by the selective growth of lymphoma cells within the lumina of vessels, especially capillaries. Two clinical variants associated with ethnogeographic origin have been described. Western patients usually present with the classical form, characterized by cutaneous involvement, as well as a higher propensity for CNS involvement; patients from Asian countries, on the other hand, tend to manifest hemophagocytic syndrome at diagnosis [7]. As is the case with DLBCL, CNS involvement portends an adverse prognosis with a median survival of less than 1 year. Anthracycline-based combination chemotherapy is commonly used; the addition of Rituximab has significantly improved clinical outcomes, with retrospective analyses showing 3 year survivals of 60% or greater in patients treated with immunochemotherapy. The addition of CNS-penetrating cytotoxics such as methotrexate and cytarabine, with consideration for whole brain radiation, is often employed in these settings with variable results [8].

The development of a secondary plasma cell neoplasm following remission from aggressive lymphoma is highly unusual. A review of our patient's investigations at initial diagnosis did not reveal any evidence of monoclonal gammopathy, bone marrow involvement, or end organ damage; the presence of a synchronous monoclonal gammopathy of undetermined significance (MGUS) or smoldering multiple myeloma at the time of first diagnosis is thus unlikely. The critical question is whether he developed 2 ontogenetically distinct de novo mature B-cell neoplasms disseminated in time and space or if his plasmacytoma arose as a clonally evolved relapse of his initial IV BLCL. Fifteen percent of patients with plasma cell neoplasms harbor t(11;14)(q13;q32) translocation, with consequent upregulation of cyclin D1, the cell cycle regulatory protein encoded by the *CCND1* gene. The reliability of immunohistochemistry in detecting this translocation is excellent [9]. The strong cyclin D1 positivity is thus robust evidence for the presence of this translocation in our patient's plasmacytoma. This specific molecular aberration also explains the PAX5 expression detected in the plasmacytoma. While more classically a marker of more immature B-cells, PAX5 expression is a recognized feature of the subset of myelomas associated with the t(11;14)(q13;q32) translocation [10]. The CNS IV LBCL was, in contrast, negative for cyclin D1. In light of the fact that cyclin D1 dysregulation, when associated with multiple myeloma, is an early event in oncogenesis, [11] this disparity argues substantively against clonal identity between the 2 tumors; it is likely that they arose as two independent primary neoplasms separated in space and time. Regrettably, we were unable to perform molecular tests such as FISH or DNA sequencing to more definitively evaluate the possibility of a common B-cell progenitor.

When evaluated, distinct synchronous or metachronous lymphoid neoplasms have been observed to have varying degrees of clonal relatedness. Approximately half of the 10–25% of patients with bone marrow involvement at the time of DLBCL diagnosis have discordant small B-cell disease in the bone marrow [12]. An exhaustive molecular evaluation of 21 such cases revealed substantive diversity; some were shown to be clonally unrelated synchronous neoplasms, others demonstrated identical genetic rearrangements suggesting clonal identity, and yet others revealed evidence of Richter's transformation from chronic lymphocytic leukemia (CLL) [13]. On the other hand, in the case of CLL and plasma cell neoplasm coincidence, a recent detailed molecular evaluation comparing the cytogenetic aberrations of these 2 malignancies in 5 patients found all evaluable cases to express features consistent with biclonality [14]. So far as we can tell, the occurrence of a plasma cell neoplasm following aggressive B-cell lymphoma, clonally related or otherwise, has not thus far been reported in humans. As noted so far, in most instances of successive lymphoid neoplasms, the initial or primary disease is an indolent lymphoma. There has been one report in the veterinary literature of a sheepdog with an aggressive B-cell lymphoma treated to complete remission with CHOP relapsing as multiple myeloma within 3 months [15]; sequencing analysis of the immunoglobulin heavy chain from the 2 tumors confirmed them to be clonally identical.

## 4. Conclusion

Our patient displayed several unique features at multiple points in his disease trajectory. He developed a very uncommon primary disease in IV LBCL, achieved satisfactory remission in the face of multiple poor prognostic factors, and developed a secondary plasma cell neoplasm, a disease more commonly associated with prior or concomitant low grade lymphomas when considered in the context of secondary lymphomas. Based on prior molecular evaluations of secondary myeloma and the disparity in cyclin D1 expression between the 2 tumors, we contend that the 2 tumors are more likely to have been clonally distinct; more extensive molecular evaluation of the tumors would be required to establish this definitively. Regardless, such a fascinating coincidence of successive rare mature B-cell neoplasms can only add to our ever growing appreciation of the prodigious complexities of malignant B-cell biology.
